# Skin Tone in Biophotonics

**DOI:** 10.1117/1.BIOS.3.2.022501

**Published:** 2026-07-01

**Authors:** Jessica Ramella-Roman, Kimani Toussaint, Muyinatu A. Lediju Bell, Chetan Patil, Matthew Keller

**Affiliations:** a Florida International University, College of Engineering and Computing, Department of Biomedical Engineering, Miami, Florida, United States; b Brown University, School of Engineering, Providence, Rhode Island, United States; c Johns Hopkins University, Whiting School of Engineering, Baltimore, Maryland, United States; d Johns Hopkins School of Medicine, Baltimore, Maryland, United States; e Aimloxy, Scranton, Pennsylvania, United States; f Armor Medical Inc., St. Louis, Missouri, United States

## Abstract

The editorial presents an overview of the BIOS special section on Skin Tone in Biophotonics.

“Skin Tone in Biophotonics,” a special section for *Biophotonics Discovery*, highlights how variation in human skin tone influences optical measurements, device performance, and clinical outcomes across biomedical imaging and sensing. Published as a series in *Biophotonics Discovery*, the collection reflects a growing recognition that skin pigmentation—particularly melanin—affects signal quality, quantification, and interpretation in ways that are only recently being systematically characterized.

Several contributions focus on photoacoustic imaging, where light absorption in the epidermis can directly affect the visibility of subsurface structures. Rasquinha et al. investigated the detectability of targets such as blood vessels using a computational model based on anatomically realistic breast tissue. By varying skin tone, target size, and reconstruction method, they show that epidermal melanin can significantly degrade image quality. Their results indicate that combining 1064 nm excitation with short-lag spatial coherence (SLSC) beamforming improves visualization across a wide range of conditions, highlighting a pathway toward mitigating skin tone–dependent bias in image reconstruction.

Complementing this modeling work, Bulthuis et al. examine the problem experimentally using a three-dimensional photoacoustic tomography setup. They introduce a hemispherical phantom incorporating both blood-mimicking fluid and pigmented skin layers to evaluate how melanin affects image quality and oximetry measurements in breast imaging. Their study documents artifacts such as spectral coloring, reflection effects, and reduced signal-to-background ratio—phenomena consistent with those observed in two-dimensional systems—while noting comparatively lower levels of clutter in the 3D configuration. The phantom design provides a controlled framework for studying these effects and may support more standardized testing of emerging imaging systems.

Other articles in the collection extend these themes to widely used sensing modalities such as pulse oximetry and photoplethysmography (PPG). Multiple studies examine how skin pigmentation alters measured oxygen saturation and signal strength, contributing to systematic errors if unaccounted for. Approaches include both experimental characterization and computational modeling, such as Monte Carlo simulations used to evaluate and correct skin tone–dependent bias. These efforts are informed by well-documented discrepancies in pulse oximetry performance, which became widely recognized during the COVID-19 pandemic. The article by Putcha et al. finds that this discrepancy in performance is likely attributed to attenuation of the red wavelength by skin pigmentation.

The collection also addresses the underlying optical properties of skin. Work on epidermal optics and tissue modeling provides a framework for understanding how parameters such as absorption, scattering, and wavelength dependence interact with melanin concentration. These studies emphasize that the magnitude and nature of skin tone effects vary across modalities, geometries, and measurement conditions, reinforcing the need for modality-specific solutions.

A related theme is the challenge of defining and quantifying skin tone itself. Contributions explore methods ranging from smartphone-based colorimetry (Burrow et al.) to more formalized spectral and typology-based approaches, reflecting the absence of consensus standards. This lack of standardization complicates comparisons across studies and limits reproducibility, particularly when evaluating device performance across diverse populations.

Across the collection, a consistent focus is the development of mitigation strategies. These include hardware approaches—such as multiwavelength sensing and polarization-sensitive detection—as well as algorithmic corrections and improved modeling of light transport in tissue. In the case of the former, a study by Jakachira et al. suggests that incorporating polarization gating into a wearable PPG sensor improves the fidelity of detected signals for red and infrared wavelengths, especially for dark skin tones. In the latter case, Chan et al. propose a pulse oximetry model that includes a melanin-correction factor, and conclude that this factor successfully reduces observed skin-tone bias. Experimental tools, including the type of tissue-mimicking phantoms described by Bulthuis et al., play a key role in validating these approaches under controlled conditions.

Taken together, the articles document a shift in biophotonics research toward explicitly addressing variability in skin optics as a central design constraint. By combining theoretical modeling, experimental validation, and measurement innovation, the collection captures the current state of the field while pointing toward more robust and inclusive optical technologies.

A list of articles is provided below for convenient reference.

• “Optical phantoms for the characterization and validation of imaging and spectroscopy: a review” (Krause et al.; doi: 10.1117/1.BIOS.3.2.022511)
• “Impact of skin pigmentation on photoacoustic tomography: a phantom study” (Balthuis et al.; doi: 10.1117/1.BIOS.3.2.022510)
• “Evaluation of a polarization-sensitive, dual-wavelength wearable photoplethysmography sensor across a range of skin tones” (Jakachira et al.; doi: 10.1117/1.BIOS.3.1.012509)
• “Effect of skin color and other skin properties on the delivered light dose in phototherapy for neonatal hyperbilirubinemia” (Dam-Vervloet et al.; doi: 10.1117/1.BIOS.2.3.032508)
• “Skin tone corrected pulse oximetry models evaluated through reflective pulsatile Monte Carlo simulations” (Chan, Rathod, and Franklin; doi: 10.1117/1.BIOS.2.3.032507)
• “Characterizing the influence of skin pigmentation on pulse oximetry” (Putcha et al.; doi: 10.1117/1.BIOS.2.3.032506)
• “What do we know about the epidermis’ optical properties and their influence on optical device functionality?” (Boonya-ananta et al.; doi: 10.1117/1.BIOS.2.3.032505)
• “Smartphone tristimulus colorimetry for skin-tone analysis at common pulse oximetry anatomical sites” (Burrow et al.; doi: 10.1117/1.BIOS.2.3.032504)
• “Effects of skin tone and adipose thickness on frequency domain near-infrared spectroscopy and diffuse correlation spectroscopy” (Gómez and Roblyer; doi: 10.1117/1.BIOS.2.1.012503)
• “Impact of skin tone on target size detectability in photoacoustic breast imaging” (Rasquinha, Gubbi, and Bell; doi: 10.1117/1.BIOS.2.1.012502)

